# Posttraumatic Stress Disorder Symptoms in Lymphoma Patients: A Prospective Study

**DOI:** 10.3389/fpsyt.2020.00201

**Published:** 2020-03-11

**Authors:** Claire Camille, Chloé Dimeglio, Antoine Yrondi, Gisèle Compaci, Emmanuelle Delmas, Mélanie Gauché, Guy Laurent, Philippe Birmes

**Affiliations:** ^1^ Toulouse NeuroImaging Center, Université de Toulouse, Inserm, UPS, Toulouse, France; ^2^ Faculté de Médecine de Toulouse, Biostatistique Informatique Médicale, Université Paul Sabatier, UMR 1027, Toulouse, France; ^3^ Departement d'Hématologie, Institut Universitaire du Cancer de Toulouse, Oncopôle, France; ^4^ CERES (Culture, Ethique, Religion et Société), Institut Catholique de Toulouse, Toulouse, France

**Keywords:** cancer, lymphoma, peritraumatic distress, peritraumatic dissociation, posttraumatic stress disorder, stressor

## Abstract

The cancer experience may be marked by repeat stressors and/or traumas. The aim of our study was to assess traumatic events in a group of patients diagnosed with lymphoma and to determine which of these contribute to the development of Post-Traumatic Stress Disorder/PTSD. Two weeks after receiving a diagnosis of lymphoma, patients were referred for an assessment of peritraumatic distress (using the Peritraumatic Distress Inventory/PDI) and peritraumatic dissociation (using the Peritraumatic Dissociative Experiences Questionnaire/PDEQ). Three months after the diagnosis, we recorded the following parameters: the patients' worst experiences, the presence of PTSD symptoms, using the PTSD CheckList/PCL, as it related to the diagnosis, and symptoms of anxiety using the Hospital Anxiety and Depression/HAD scale and of depression using the Beck Depression Inventory/BDI-II. The study recruited 129 patients, with a mean age of 46 years (SD = 17.3); 70 (54%) men, 87 (67.5%) with Non-Hodgkin's lymphoma, and 42 with Hodgkin's lymphoma. Two weeks after the diagnosis, 49% of patients reported peritraumatic distress, and 20% peritraumatic dissociation, during or immediately after being informed of the lymphoma diagnosis. Three months after the diagnosis, the severity of PTSD symptoms was evaluated. At this stage none of the patients suffered PTSD, but 29 (23%) individuals exhibited partial PTSD: 13.4% correlated it to receiving the lymphoma diagnosis, 8% to telling family members, and 1.6% to adverse effects. Peritraumatic distress and dissociation as a result of receiving a lymphoma diagnosis, as well as anxiety and a mucositis within the first 3 months post-diagnosis, were factors that were significantly associated with PTSD symptoms, accounting for 35.8% in PTSD symptom load. Our study reveals that clinicians should assess the impact of a number of stressors, which are risk factors for PTSD symptoms, starting from the time point of the initial lymphoma diagnosis.

## Introduction

Being diagnosed and treated for cancer may be a traumatic experience associated with heightened distress (1). Disorders which are precipitated by specific stressful and potentially traumatic events are included in the spectrum of Trauma and Stress-Related Disorders, and encompass both Adjustment Disorders (ADs) and PTSD (1). Investigators have on many occasions reported stress and trauma-related symptoms in cancer survivors such as intrusive memories, avoidance behaviors and negative alterations in cognition and mood, as well as hyperarousal. PTSD is a clinically-significant condition with symptoms persisting for longer than one month after exposure to a traumatic event and cause significant distress and impairment in social, occupational and other important functional areas. PTSD criteria include: intrusion symptoms (e.g., recurrent, involuntary, and intrusive distressing memories of the traumatic event); persistent avoidance of stimuli associated with the traumatic event (e.g., avoidance of or efforts to avoid distressing memories, thoughts, or feelings about or closely associated with the traumatic event); negative alterations in cognitions and mood associated with the traumatic event (e.g., persistent and exaggerated negative beliefs or expectations about oneself, others, or the world); and marked alterations in arousal and reactivity associated with the traumatic event (e.g., hypervigilance) (2).

Following a diagnosis of cancer, the incidence of full-blown PTSD ranges from 3%–4% in recently diagnosed early stage patients, and up to 35% in patients evaluated after cancer treatment (3). A meta-analysis (4) reviewed 25 studies (21 of these studies involved breast cancer patients) of cancer-related PTSD in a total of 4,189 adult cancer survivors. Studies based on patients' self-reported PTSD symptoms yielded prevalence estimates of clinically significant symptoms ranging from 7.3% (95% CI 4.5–11.7; ten studies) to 13.8% (9.5–19.6; 11 studies). A large longitudinal study of adult lymphoma survivors (5) found that 12% exhibited PTSD symptoms that resolved over a 5-year time period. More than one third of patients reported persistent (18%) or exacerbated (19%) PTSD symptoms over a 5-year time frame. In survivors of Hodgkin's lymphoma, Varela et al. (6) showed that a large proportion of survivors (35.2%) met the criteria for partial PTSD. The female gender might therefore be a risk factor for PTSD symptoms. Smith et al. (7) studied post-traumatic stress outcomes in survivors of Non-Hodgkin's Lymphoma and reported that patients suffering from PTSD were younger than patients not exhibiting PTSD.

The exposure to actual death or the threat of death during a terrorist attack is irrefutable. The suddenness of the impact is comparatively more moderate for the diagnosis of a serious illness. When a patient is faced with the diagnosis of a lymphoma with a 25% survival rate at 5 years, the traumatic exposure nevertheless, seems very real. However, trauma is not only associated with distress, but also with posttraumatic psychological growth (8). The typical cancer diagnosis experience which often begins with an anomaly detected during self-examination, an anomalous laboratory test, screening procedure, routine imaging, or clinical examination, followed by the news that one has a life-threatening illness, can be shocking. Moreover, treatment of malignant disease might entail a series of acute and prolonged challenging stages, including surgery, chemotherapy, radiation, immunotherapy, and hormonal therapy and their corresponding side-effects (9).

The most difficult aspect of assessing trauma and stress-related mental disorders in cancer settings is therefore precisely when to evaluate the patient. The cancer experience may be marked by repeat stressors and/or traumas. This means that an individual may exhibit symptoms at any point starting from diagnosis and extending throughout treatment (3), with the emotional impact of being informed of the diagnosis representing the first potentially traumatic event. The aim of our study was to assess the initial stressors or traumatic events in a group of patients diagnosed with lymphoma and to identify which of these events are specifically correlated to the development of PTSD symptoms. Our primary hypothesis was that the emotional distress experienced at the time of the cancer diagnosis was a significant risk factor in the subsequent development of PTSD symptoms.

## Methods

### Subjects and Procedure

Patients were recruited from the University Cancer Institute of Toulouse, France. The study was approved by the ethics committee for research on human subjects at the Toulouse University Hospital. Participants gave their written informed consent after receiving a comprehensive explanation of the procedures. The inclusion criteria were: age≥18 years; a current Non-Hodgkin's lymphoma (NHL) or Hodgkin's lymphoma (HL) diagnosis. The exclusion criteria were: age < 18 years, tutelage, trusteeship, or legal protection, severe cognitive impairment and a lack of French language proficiency. Two weeks (T1) after being diagnosed with lymphoma, patients were referred for an assessment of the emotional and cognitive impact of the diagnosis and to record their previous psychiatric medical history (**Table 1**). The following parameters were recorded three months after the initial lymphoma diagnosis (T2): the patients' worst experiences, the presence of PTSD symptoms related to this event, and anxiety and depression symptoms. The data assessment was face-to-face at the initial assessment and face-to-face at follow-up. The study identified 190 patients diagnosed with lymphoma, between January 2015 and June 2016. Of these, 24 patients opted not to participate in the study (12.6%) and 36 did not meet the inclusion criteria (18.9%). Ultimately 130 patients took part in the study (68%). One patient died during the course of the study, but no other patients were lost to the study.

**Table 1:** Sample characteristics.

Demographic variables	N	%	Mean	Standard deviation	Max	min
**Gender**	129					
Woman	59	46%				
Man	70	54%				
**Age**			46.1	17.3	79	18
**Employment status**						
Employed	7	5.4%				
Unemployed	8	6.2%				
Retired	18	14%				
Student	18	14%				
Sick leave	78	60.4%				
**Profession**						
Farmer	5	3.9%				
Artisan, business manager	7	5.4%				
Executive	9	7%				
Independent	6	4.6%				
Employee	41	31,8%				
Laborer	4	3.1%				
Others	57	44.2%				
**Education level**						
Primary	4	3.1%				
Certificate	3	2,3%				
French national diploma for year 10/9th grade students	29	22,3%				
French national academic qualification obtained at the end of secondary education	26	20%				
Tertiary education	68	52.3%				
**Smoker**						
Yes	20	15.6%				
No	109	84.4%				
**Type of hemopathy**						
HL	42	32.5%				
NHL	87	67.5%				
**MDE history**						
Past	4	3.1%				
Current	6	4.6%				
**GAD history (current)**	23	17.8%				
**Peritraumatic distress**	63	49%				
**Peritraumatic dissociation**	27	20%				
**Partial PTSD (T2)**	29	23%				
**non clinical level**	100	77%				
**Depressive symptoms (T2)**						
**moderate**	5	4%				
**mild**	1	1%				
**non clinical level**	123	95%				
**Anxiety symptoms (T2)**						
**mild**	35	27%				
**major**	11	8%				
**non clinical level**	83	65%				

sMDE, major depressive episode; GAD, generalized anxiety disorder; PTSD, post-traumatic stress disorder.

### Clinical Assessment

#### Two Weeks Post-Diagnosis (T1)

The level of emotional distress (helplessness, fear, horror) upon receiving the diagnosis of lymphoma was assessed using the Peritraumatic Distress Inventory/PDI validated in French, a 13-item self-report questionnaire assessing the level of emotional distress experienced during a traumatic event (10). Each item was scored on a 5-point Likert scale from 0 (not true) to 4 (extremely true). The total score was obtained by adding up the individual responses for all items and ranged from 0 to 52, with higher scores indicating increased peritraumatic distress (Cronbach's α of.84 in our sample). Subjects who rated items 1 (I felt helpless to do more), 4 (I felt afraid for my safety), or 10 (I was horrified by what happened) as at least 3 (very true) were considered to have suffered clinically relevant peritraumatic distress (10, 11).

The degree of cognitive dissociation (moments of losing track of time, the event seeming unreal, etc.) when receiving the diagnosis of lymphoma was assessed using the Peritraumatic Dissociative Experiences Questionnaire/PDEQ validated in French (12), a 10-item self-report questionnaire assessing the degree of cognitive dissociation experienced during trauma. Each item was scored on a 5-point Likert scale ranging from 1 (not at all true) to 5 (extremely true). The sum of all items provided a total score ranging from 10 to 50, with higher scores indicating increased peritraumatic dissociation (Cronbach's α of.90 in our sample). Subjects with scores ≥ 22 were considered to have clinically significant levels of peritraumatic dissociation.

A psychiatric medical history was assessed using the Mini International Neuropsychiatric Interview (MINI 5.0.0) (13). This structured diagnostic interview evaluates the main psychiatric axis I, DSM-IV disorders. In our study he MINI was only used for major depressive episode (MDE) and generalized anxiety disorder (GAD) symptoms.

#### Three Months Post-Diagnosis (T2)

The PTSD CheckList-Specific/PCL-S validated in French (14), was used to assess PTSD symptoms. During the course of this study no validated French scale, corresponding to the DSM-5 criteria, was available to evaluate PTSD symptoms. The PCL-S is a 17-item self-reporting tool. Each item is scored on a 5-point Likert scale (1 ‘‘not at all'' to 5 ‘‘very often''). Total scores ranged from 17 to 85, with higher scores reflecting increased levels of PTSD symptoms. Scores > 44 reflect conditions justifying clinical attention (“probable PTSD”) (Cronbach's α of.91 in our sample). Symptomatic individuals who failed PTSD diagnostic criteria were categorized as exhibiting “partial” (“subthreshold” or “subsyndromal”) PTSD. A widely accepted strategy for assigning a partial diagnosis of PTSD is the requirement for at least one intrusion, one avoidance, and one hyperarousal symptom (15).

To assess depression symptoms, we used the Beck Depression Inventory/BDI-II scale (16). The BDI is one of the most easily implemented and most widely used tools for measuring subjective aspects of depression. Its 22 items are graded from 0 (absence of symptoms) to 3 (severe symptoms). The BDI-II gives an overall score of the intensity of the depressive syndrome ranging from 0 to 66 (Cronbach's α of.89 in our sample), with scores from 0–13 equating to minimal depression, 14–19 mild depression, 20–28 moderate depression, and scores of 29–63 identifying severe depression.

The Hospital Anxiety and Depression/HAD scale was used to evaluate anxiety (17). This scale is used for routine assessment in the Hematology Department. Our analysis was restricted to the seven items assessing anxiety. Each item was rated by the patient on a four-point (0–3) response category scale, with possible anxiety scores ranging from 0 to 21. With a score of 11 or higher indicating the potential presence (“caseness”) of the state.

Asthenia and nausea are included in the routine assessment (by nurses) of lymphoma patients in the oncology department. In our study, this assessment was scored as a yes/no.

### Analysis

Variables associated with PTSD status were evaluated using univariate analyses. Chi-squared and Fisher's exact tests, Student's t test or the Mann-Whitney U test were used as appropriate (data shown in **Supplementary Data**). We then used a logistic regression model to identify independent variables associated with PTSD status, by analyzing variables with a P value < .20 after the univariate analysis. We subsequently added gender and age to account for any potential confounding factors, and then also the mucositis variable, which was statistically associated with the outcome in a previously conducted study assessing a shorter period of time than our study (18). Statistical significance was set at P < .05. We tested interactions between all variables included in the final model: for each potential interaction we performed a model with interaction and a model without any term of interaction. We then performed a likelihood-ratio test to compare the two models. For each potential interaction, all the p-values of the likelihood-ratio tests were not significant.”

## Results

Our study recruited 129 lymphoma patients, with a mean age of 46 years (SD = 17.3), over a period of 18 months (**Table 1** sample characteristics). These included 70 (54%) men and 59 women (46%), 87 patients had been diagnosed (67.5%) with Non-Hodgkin's lymphoma, and 42 with Hodgkin's lymphoma. All patients were treated according to the French therapeutic standard for HL and NHL. All patients took part in the Navigator procedure during their initial treatment. The Navigator procedure is a service offered to lymphoma patients treated as outpatients that involves the patient being contacted by a nurse *via* telephone twice a week. (19). The study included 4 (3.1%) patients with a prior medical history of depression, 6 (4,6%) with a current diagnosis of depression and 23 (17.8%) with a current diagnosis of generalized anxiety disorder.

Two weeks after the diagnosis (T1) 49% of patients reported peritraumatic distress, and 20% reported peritraumatic dissociation during or immediately after being informed of their lymphoma diagnosis. At three-months post diagnosis, the second assessment (T2) showed that the worst events experienced during the first 3 months were: being informed about the lymphoma diagnosis (64%); adverse effects: adverse drug reaction, lumbar puncture (23%); and communicating the lymphoma diagnosis to family members (13%).

Three months after the diagnosis, none of the patients in the study were confirmed to have full PTSD, but 29 (23%) expressed clinically relevant PTSD symptoms (partial PTSD): 13.4% related it to receiving the diagnosis of lymphoma, 8% to telling family members, and 1.6% to adverse effects. Fifteen (15.5%) patients exhibited mild depression symptoms.

Patients who expressed clinically relevant PTSD symptoms (partial PTSD) were more likely to be smokers, to have previously reported peritraumatic distress or dissociation and to have exhibited symptoms of anxiety and mild depression (**Table 2**). Peritraumatic distress and dissociation when receiving a diagnosis of lymphoma, with anxiety and a mucositis within the first 3 months post-diagnosis, accounted for 35.8% of the model's variance (**Table 3**).

**Table 2:** Variables assessed two weeks post-diagnosis (T1) associated with partial PTSD after receiving a lymphoma diagnosis.

	No Partial PTSD		Partial PTSD		χ2	p
	(n = 100)	(77%)	(n = 29)	(23%)		
Smokers	8	8%	12	41%	7.64	0.02
Peritraumatic dissociation^1^	7	7%	20	68%	17.47	<0.000
Peritraumatic distress^2^	40	40%	23	79%	25.54	<0.000
Mild depression^3^	6	6%	14	48%	11.25	0.02
Anxiety^4^	2	2%	11	37%	26.45	<0.000
Asthenia	52	52%	16	55%	8.47	0.07
Nausea	37	37%	15	51%	6.35	0.17
Mucositis	13	13%	6	20%	6.79	0.34

1 Peritraumatic Dissociative Experiences Questionnaire total score ≥22.

2 Peritraumatic Distress Inventory item helplessness or item fear or item horror with a score of 3=very true or 4 = extremely true.

3 Beck Depression Inventory-II total score 14-19.

4 Anxiety total score of the Hospital Anxiety and Depression scale ≥8.

**Table 3:** Summary of logistic regression analysis of variables predicting partial PTSD after receiving a lymphoma diagnosis.

	Odds ratio	Standard error	95% Confidence Interval		*P* value
*N = 129*					
Analysis PCL-S PART content (*R^2^* = 0.358)					
Peritraumatic distress	1.14	0.05	1.03	1.27	0.007
Anxiety	1.18	0.7	1.04	1.34	0.01
Mucositis	0.42	0.16	0.20	0.90	0.027
Peritraumatic dissociation	1.14	0.06	1.03	1.27	0.011

## Discussion

In contrast to other studies (5, 6), we did not detect any cases of full PTSD in a group of 129 patients diagnosed with lymphoma within three months post-diagnosis. This does not however necessarily mean that patients do not feel distressed. Several studies identified cancer as a traumatic stressor with the perception of a threat to life more commonly reported than responses involving fear, helplessness and horror (9). We did find that 23% of patients exhibited partial PTSD where symptoms cause clinically significant distress but do not meet the full criteria for PTSD. The presence of at least one intrusion symptom led us to call this disorder partial PTSD, rather than adjustment disorder. In our study the prevalence of depression was low and the current GAD diagnoses may potentially be biased by patients' current lymphoma related health concerns.

In another study of patients with NHL, 7.4% were suspected to have full PTSD and 9% partial PTSD (7). However, these PTSD case were related to the “cancer” in general and did not evaluate any specific event (9). Our study also identified the nature of the biggest stressors in the context of a “cancer experience”. Being informed of the cancer diagnosis, informing relatives and adverse effects were the most frequently cited events by patients. A review of the literature had previously reported that diagnosis and treatment were the most traumatic events, but had not evaluated additional details (20).

We showed that peritraumatic distress and dissociation during or immediately following the diagnosis of HL or NHL were risk factors for PTSD symptoms. Peritraumatic distress and dissociation at the time of receiving a diagnosis of lymphoma may be early risk factors for trauma and stressor related symptoms. In our study, 49% of patients reported peritraumatic emotional distress and 20% peritraumatic cognitive dissociation. Peritraumatic distress has been well accepted as an early risk factor for the development of PTSD for a number of years now (11). Although this is the first time that emotional distress during or immediately after being informed of a cancer diagnosis has been assessed using the Peritraumatic Distress Inventory, other studies have assessed emotional distress around a cancer diagnosis and PTSD at a later time point. For example, cancer-related acute stress disorder has been assessed in a previous study, but appeared to have modest predictive power for later development of cancer-related PTSD (1). A study conducted in 92 patients with a variety of cancers showed that 31.5% of patients developed peritraumatic dissociation symptoms, although peritraumatic distress was not assessed and peritraumatic dissociation was assessed retrospectively (21).

Our results are in agreement with a meta-analysis (22) which showed that cancer-related PTSD symptoms were positively correlated with depression, anxiety, and overall distress. As for the perceived level of mucositis three months after being informed of the diagnosis, our results concur with a (18) study which reported a positive correlation between the occurrence of PTSD and the intensity and duration of mucositis.

The small sample size of our study may have precluded the observation of any full PTSD cases. However, using the concept of partial PTSD in research and practice seems a useful distinction (15). Moreover using the PCL-S is not diagnostic, but rather assesses symptom severity. Most comparable studies used the PCL without questioning the subject specifically about an index event, but about “cancer” in general. This approach may indeed overestimate cases of cancer-related PTSD (7). Generally, PTSD symptom onset is described a few days after the traumatic event and many patients recover without requiring treatment within months/years of the triggering event (50% natural remission by 2 years) (23). With a longer follow-up period the number of individuals that are suspected to have PTSD may actually decrease.

Our study demonstrates the importance of evaluating psychological responses as soon as the patient is informed about the cancer diagnosis. Although we did not observe the same proportion of PTSD cases as reported in previous studies of lymphoma patient groups, we did confirm that many patients are affected by trauma- and stressor-related symptoms. We also identified the principal stressors for which significant peritraumatic emotional distress may be a major risk factor and for the rapid development of these kinds of cancer-related symptoms. Potential stressors and traumatic events are numerous, occurring as early as the first few weeks following the lymphoma diagnosis. Clinicians should assess the impact of such stressors, starting from when the patient is informed of the lymphoma diagnosis. The accuracy of diagnosing different types of cancer-related stress disorders has a direct impact on determining the most beneficial and appropriate treatment options for the individual patient (1). When a cancer diagnosis is given, the immediate identification of various responses to stress and to emotional, physiological, and cognitive distress may lead to adopting interventional approaches such as the Brief Dyadic Therapy or Self-guided Internet Based Intervention. Recent evidence has emerged to support the benefits of this type of approach for the prevention of PTSD symptoms in adults (24).

## Data Availability Statement

The datasets generated for this study are available on request to the corresponding author.

## Ethics Statement

The studies involving human participants were reviewed and approved by the ethics committee for research on human subjects at the Toulouse University Hospital, France. The patients/participants provided their written informed consent to participate in this study

## Author Contributions

Conception and design of the work (CC, GC, ED, GL, and PB), data analysis and interpretation (CC, CD, and PB), drafting the article (CC, PB), critical revision of the article (AY, MG, GL, and PB), and final approval of the version to be published (CC, CD, AY, GC, ED, MG, GL, and PB).

## Conflict of Interest

The authors declare that the research was conducted in the absence of any commercial or financial relationships that could be construed as a potential conflict of interest.
