# Supplementary material for: Posttraumatic Stress Disorder Symptoms in Lymphoma Patients: A Prospective Study
Source: Front Psychiatry. 2020 Mar 11;11:201. doi: 10.3389/fpsyt.2020.00201 (PMC7078238; doi:10.3389/fpsyt.2020.00201)
Supplement: Supplementary file 1 [file DataSheet_1.docx]

Bivariate analysis at 3-months post-diagnosis

Three months after being informed of their diagnosis, 129 patients responded to the PCL, BDI-II and ADH questionnaires.

Of these 129 participants, 29 (23%) were found to exhibit partial PTSD symptoms.

Partial PTSD and smoker (yes/no)

Partial PTSD and adverse effects of chemotherapies

Summary table of variables associated with PTSD three months after being informed of the lymphoma diagnosis (χ2 or Fisher's exact test)

|  | Without PTSD | | Partial PTSD | | χ^2^  or Fisher’s exact test (p) |
| --- | --- | --- | --- | --- | --- |
| Smoker | 8 | 8% | 12 | 41% | 0.02 |
| Asthenia | 52 | 52% | 16 | 55% | 0.03 |

Partial PTSD and peritraumatic responses

Summary table of variables associated with partial PTSD three months after being informed of the lymphoma diagnosis (χ2 or Fisher's exact test)

|  | N=129 | Mean | Standard deviation | T | P |
| --- | --- | --- | --- | --- | --- |
| Peritraumatic Distress^1^ | 129 | 8.50 | 7.07 | -7.01 | 0.00 |
| Peritraumatic Dissociation^2^ | 129 | 15.06 | 6.09 | -7.09 | 0.00 |
| Depression^3^ | 129 | 6.33 | 4.46 | -3.25 | 0.001 |
| Anxiety^4^ | 129 | 5.67 | 4.54 | -5.72 | 0.00 |

^1^ Peritraumatic Dissociative Experiences Questionnaire

^2^ Peritraumatic Distress Inventory

^3^ Beck Depression Inventory-II

^4^ Hospital Anxiety and Depression scale anxiety score

### Logistic regression three months after being informed of the diagnosis.

### We sought to identify the underlying factors explaining the symptoms of PTSD bias three months after being informed of the diagnosis. To do so, the initial logistic regression model included variables associated with outcome from the bivariate analyses with a threshold of less than or equal to 20%, i.e. :

- Nausea (categorical variable)
- Asthenia (categorical variable)
- Smoker (binary variable)
- Peritraumatic dissociation (binary variable)
- Peritraumatic distress (binary variable)
- Depression (categorical variable with BDI-II)
- Anxiety (categorical variable with HAD)

We then proceeded by eliminating, the variables least associated with the PTSD bias (p-value criterion) in successive logistic regressions, to eventually obtain a final parsimonious model.

Logistic Regression

|  | Odds ratio | Standard Error | 95% CI | | *P* |
| --- | --- | --- | --- | --- | --- |
| *N=129* | | | | | |
| Analyse partial PTSD (*R^2^* =0.3580) | | |  | | |
| Peritraumatic Distress | 1.14 | 0.05 | 1.03 1.27 | | 0.007 |
| Anxiety | 1.18 | 0.7 | 1.04 | 1.34 | 0.010 |
| Mucite | 0.42 | 0.16 | .20 | 0.90 | 0.27 |
| Peritraumatic Dissociation | 1.14 | 0.06 | 1.03 | 1.27 | 0.011 |
